# Local and Systemic Therapy of Recurrent Medulloblastomas in Children and Adolescents: Results of the P-HIT-REZ 2005 Study

**DOI:** 10.3390/cancers14030471

**Published:** 2022-01-18

**Authors:** Christine Gaab, Jonas E. Adolph, Stephan Tippelt, Ruth Mikasch, Denise Obrecht, Martin Mynarek, Stefan Rutkowski, Stefan M. Pfister, Till Milde, Olaf Witt, Brigitte Bison, Monika Warmuth-Metz, Rolf-Dieter Kortmann, Stefan Dietzsch, Torsten Pietsch, Beate Timmermann, Ronald Sträter, Udo Bode, Andreas Faldum, Robert Kwiecien, Gudrun Fleischhack

**Affiliations:** 1 Department of Pediatrics III, Center for Translational Neuro- and Behavioral Sciences (CTNBS), University Hospital of Essen, Hufelandstrasse 55, 45147 Essen, Germany; christine.gaab@uk-essen.de (C.G.); jonas.adolph@uk-essen.de (J.E.A.); stephan.tippelt@uk-essen.de (S.T.); ruth.mikasch@uk-essen.de (R.M.); 2 Department of Pediatric Hematology and Oncology, Center for Obstetrics and Pediatrics, University Medical Center Hamburg-Eppendorf, 20251 Hamburg, Germany; d.obrecht@uke.de (D.O.); m.mynarek@uke.de (M.M.); s.rutkowski@uke.de (S.R.); 3 Mildred Scheel Cancer Career Center HaTriCS4, University Medical Center Hamburg-Eppendorf, 20246 Hamburg, Germany; 4 Hopp Children’s Cancer Center Heidelberg (KiTZ), 69120 Heidelberg, Germany; s.pfister@kitz-heidelberg.de (S.M.P.); t.milde@kitz-heidelberg.de (T.M.); o.witt@kitz-heidelberg.de (O.W.); 5 Division of Pediatric Neuro-Oncology, German Cancer Consortium/DKTK and German Cancer Research Center (DKFZ), 69120 Heidelberg, Germany; 6 Department of Pediatric Oncology and Hematology, Heidelberg University Hospital, 69120 Heidelberg, Germany; 7 Clinical Cooperation Unit Pediatric Oncology, German Cancer Research Center (DKFZ), German Consortium for Translational Cancer Research (DKTK), 69120 Heidelberg, Germany; 8 Department of Neuroradiology, University Hospital Augsburg, 86156 Augsburg, Germany; brigitte.bison@uk-augsburg.de; 9 Institute of Diagnostic and Interventional Neuroradiology, University of Würzburg, 97080 Würzburg, Germany; warmuth_m@ukw.de; 10 Department of Radio-Oncology, University of Leipzig, 04103 Leipzig, Germany; rolf-dieter.kortmann@medizin.uni-leipzig.de (R.-D.K.); stefan.dietzsch@medizin.uni-leipzig.de (S.D.); 11 Department of Particle Therapy, University Hospital Essen, West German Proton Therapy Centre Essen (WPE), West German Cancer Center (WTZ), German Cancer Consortium (DKTK), 45147 Essen, Germany; beate.timmermann@uk-essen.de; 12 Institute of Neuropathology, DGNN Brain Tumor Reference Center, University of Bonn, 53127 Bonn, Germany; torsten.pietsch@ukbonn.de; 13 Department of Pediatric Hematology and Oncology, University Hospital Münster, 48129 Münster, Germany; ronald.straeter@ukmuenster.de; 14 Department of Pediatric Hematology and Oncology, University Hospital Bonn, 53127 Bonn, Germany; udo.bode@ukbonn.de; 15 Institute of Biostatistics and Clinical Research, Westfälische Wilhelms-Universität Münster, Schmeddingstraße 56, 48149 Münster, Germany; faldum.andreas@ukmuenster.de (A.F.); robert.kwiecien@ukmuenster.de (R.K.)

**Keywords:** medulloblastoma, refractory, recurrent, children, chemotherapy, surgery, radiotherapy, re-irradiation, intraventricular therapy

## Abstract

**Simple Summary:**

A medulloblastoma recurrence is usually associated with an unfavorable prognosis. The German P-HIT-REZ 2005 Study gathered data from patients with relapsed medulloblastomas treated in different, non-randomized therapy arms dependent on preconditions of the patients (previous treatment, comorbidities, relapse pattern), the decision of treating physicians, and the patients’/parents’ choice. A total of 93 evaluable patients with refractory or relapsed medulloblastoma were enrolled. The main aim of this study was to analyze the impact of patient and disease characteristics as well as local and systemic therapies on post-relapse progression-free (PFS) and overall survival (OS). In multivariate analysis, a short time until the first recurrence (<18 months) was the strongest predictor for a worse PFS and OS, which was mainly associated with molecular subgroup 3. Metastatic disease, at relapse, only had a significant impact on OS. Re-biopsy, at relapse, is highly recommended to investigate the histopathological and molecular genetic tumor characteristics and to exclude a secondary malignancy.

**Abstract:**

Recurrent medulloblastomas are associated with survival rates <10%. Adequate multimodal therapy is being discussed as having a major impact on survival. In this study, 93 patients with recurrent medulloblastoma treated in the German P-HIT-REZ 2005 Study were analyzed for survival (PFS, OS) dependent on patient, disease, and treatment characteristics. The median age at the first recurrence was 10.1 years (IQR: 6.9–16.1). Median PFS and OS, at first recurrence, were 7.9 months (CI: 5.7–10.0) and 18.5 months (CI: 13.6–23.5), respectively. Early relapses/progressions (<18 months, *n* = 30/93) found mainly in molecular subgroup 3 were associated with markedly worse median PFS (HR: 2.34) and OS (HR: 3.26) in regression analyses. A significant survival advantage was found for the use of volume-reducing surgery as well as radiotherapy. Intravenous chemotherapy with carboplatin and etoposide (ivCHT, *n* = 28/93) showed improved PFS and OS data and the best objective response rate (ORR) was 66.7% compared to oral temozolomide (oCHT, *n* = 47/93) which was 34.8%. Intraventricular (*n* = 43) as well as high-dose chemotherapy (*n* = 17) at first relapse was not related to a significant survival benefit. Although the results are limited due to a non-randomized study design, they may serve as a basis for future treatment decisions in order to improve the patients’ survival.

## 1. Introduction

Approximately one-third of all patients diagnosed with medulloblastoma in childhood and adolescence develop a progression or a relapse during and after first-line treatment [1,2,3]. Primary medulloblastoma is currently treated with maximal safe resection, chemotherapy, and cranio-spinal irradiation (CSI) in patients old enough to tolerate CSI [4,5]. While primary therapy is well established and follows distinct guidelines, therapy at recurrence is not standardized [6]. Current curative strategies for relapse therapy include resection, re-irradiation, conventional and high-dose chemotherapy regimens, and metronomic antiangiogenic multi-agent therapy [3,7,8]. Despite these aggressive treatment approaches, patients with recurrent medulloblastomas have a poor prognosis with long-term overall survival rates less than 10%. In early stages, disseminated and multiple relapses require palliative therapy options in order to retain a good quality of life as long as possible [5,9,10].

Studies investigating the efficacy of different therapeutic modalities and chemotherapy regimens in medulloblastoma relapses have mostly investigated small patient cohorts and have shown dismal results so far [11,12,13]. The HIT-REZ 2005 Study was designed on the experience and results of the HIT-REZ 97 Study that was the first national German multicenter trial for the treatment of refractory and relapsed ependymomas, medulloblastomas, pineoblastomas, and CNS primitive neuroectodermal brain tumors (CNS-PNETs) in children, adolescents, and young adults [11]. The HIT-REZ 2005 Study (NCT00749723) consisted of three parts: (1) the P-HIT-REZ 2005 Study—a trial for the treatment of relapsed CNS-PNETs, medulloblastomas, and pineoblastomas, (2) the E-HIT-REZ 2005 Study—a trial for the treatment of relapsed ependymomas and (3) a Phase II-Window-Study: intraventricular therapy with etoposide in neoplastic meningitis in relapsed CNS-PNETs, medulloblastomas, and ependymomas with subarachnoid tumor manifestation. In this analysis we only report the treatment of refractory and recurrent medulloblastoma in the P-HIT-REZ 2005 Study.

**Figure 1:**
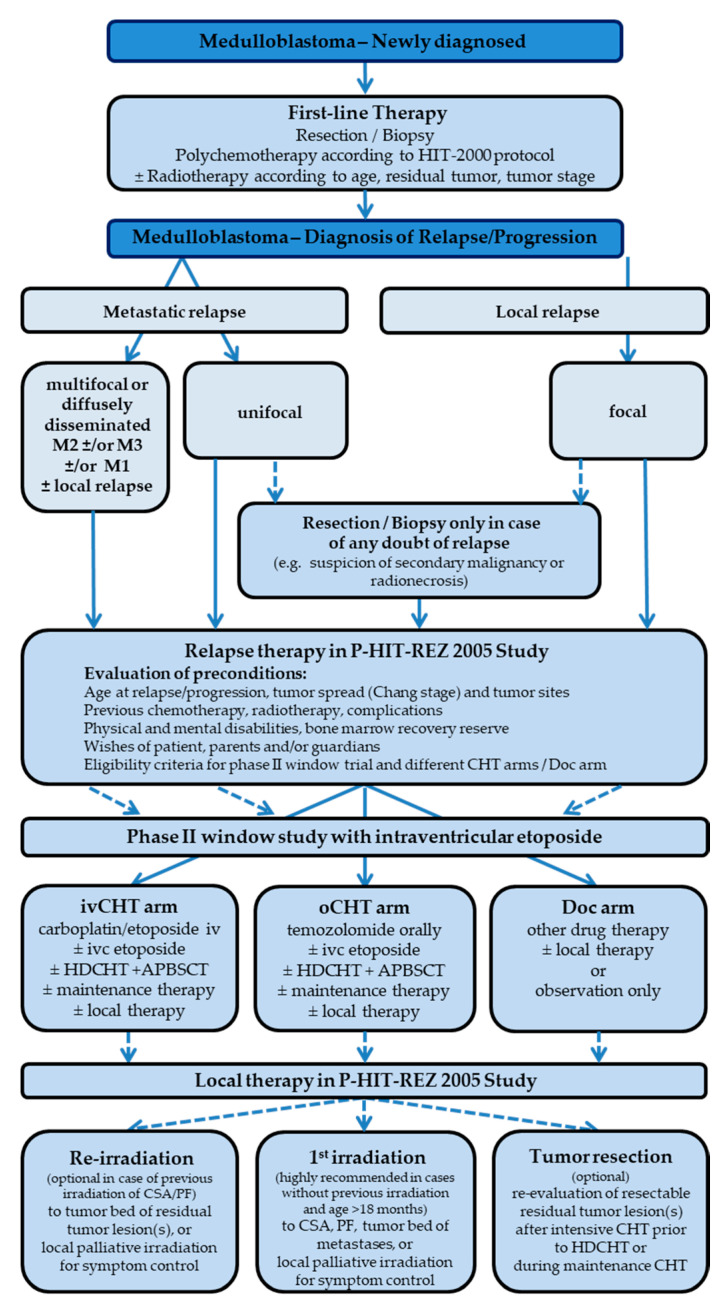
Treatment algorithm in relapsed/refractory medulloblastoma in the P-HIT-REZ 2005 Study (CHT: chemotherapy; ivCHT: intravenous chemotherapy; oCHT: oral chemotherapy; Doc: documentation; ivc: intraventricular; HDCHT: high-dose chemotherapy; APBSCT: autologous peripheral stem cell transplantation; CSA: craniospinal axis; PF: posterior fossa).

**Figure 2:**
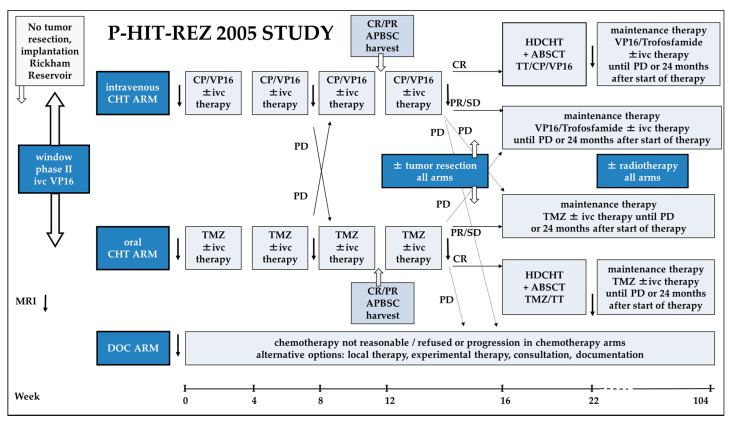
Therapy flowchart of the P-HIT-REZ 2005 Study. CHT: chemotherapy; HDCHT: high-dose chemotherapy; Doc: documentation; APBSC(T): autologous blood stem cell (transplantation); TT, thiotepa; TMZ: temozolomide; CP: carboplatin; VP16: etoposide; ivc: intraventricular; CR: complete remission; PR: partial response; SD: stable disease; PD: progressive disease.

With the availability of alternative chemotherapy approaches, such as temozolomide, and new forms of therapy, such as intraventricular therapy with etoposide, new therapeutic options should be explored. The main objective of the P-HIT-REZ 2005 Study was the evaluation of the efficacy of known systemic chemotherapy with carboplatin/etoposide compared with the newly available temozolomide by enrolling patients into specific study arms (Figure 1 and Figure 2). The allocation to the chemotherapy arms depended on the individual course of disease, on previous treatment modalities and complications, comorbidities, and based on the decision of the treating physician and the patients’ and parents’ choice. Here we report the results of the response and survival analysis within the P-HIT-REZ 2005 Study and the impact of patients’ and disease characteristics as well as of the different local and systemic therapy modalities.

## 2. Materials and Methods

### 2.1. Clinical Trial

**Figure 3:**
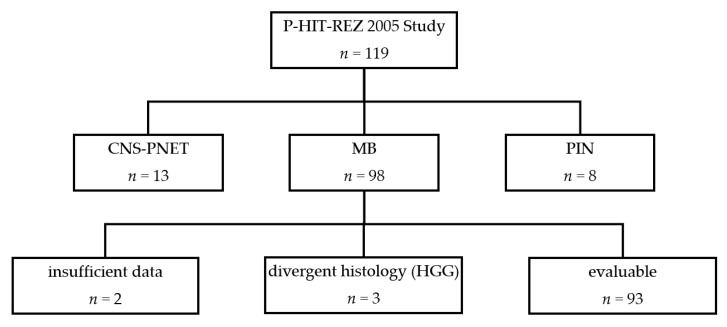
Eligible and evaluable patients in the P-HIT-REZ 2005 Study. (MB: medulloblastomas; CNS-PNET: central nervous system primitive neuroectodermal tumors; PIN: pineoblastomas; HGG: high grade glioma).

Patients suffering from a relapsed or refractory medulloblastoma enrolled into the P-HIT-REZ 2005 Study were included in this analysis. Patients were excluded from our analysis in the case of insufficient data about first recurrence/progression, the suspected recurrence histologically being confirmed as secondary malignancy, or if retrospective evaluation at relapse suggested a non-medulloblastoma entity in the central neuropathological and/or molecular analysis (Figure 3).

#### 2.1.1. Eligibility Criteria

Eligibility criteria were histologically confirmed medulloblastomas diagnosed according to the guidelines of the WHO classification of tumors of the CNS 2000 and 2007 [14] at either initial diagnosis or relapse/progression, and a centrally reviewed MRI detecting recurrence or progression. Patients aged between 3 months and 30 years at recurrence and with measurable lesion in MRI and/or detectable tumor-cells in the cerebrospinal fluid (CSF) at first or multiple relapses were eligible. Additionally, if either the enrollment in the intravenous or oral systemic therapy arms was planned, the following inclusion criteria had to be fulfilled: a life expectancy of at least 8 weeks, an ECOG of at least 3 or a Karnofsky/Lansky Performance Status Score of at least 40%, no overt cardiovascular, pulmonary, renal, or hepatic disease, no clinically significant electrolyte imbalances and no severe neurological disease that would prevent an accurate assessment of therapy tolerance. If systemic treatment involving carboplatin was planned, audiometry before the first block of chemotherapy had to exclude hearing loss in the 2–4 kHz range of either >30 dB for standard-dose carboplatin or >60 dB for high-dose carboplatin, respectively.

There were additional eligibility criteria for inclusion of patients in the phase-II-window trial and for receiving simultaneous intraventricular etoposide to the systemic chemotherapy in the ivCHT and oCHT arm: a relapse with subarachnoid metastatic or subarachnoid local disease manifestation and/or positive CSF cytology (M1 stage) without any threatening, severe neurological symptoms caused by any concurrent parenchymal tumor lesion, no signs of elevated intracranial pressure, no disturbances of CSF circulation and/or CSF resorption making a cerebral shunt permanently necessary, and presence of an inserted Ommaya or Rickham Reservoir. Prior to the intraventricular therapy, a DTPA-CSF flow scintigraphy was highly recommended to rule out any relevant disturbance of CSF circulation.

#### 2.1.2. Study Design

The P-HIT-REZ 2005 Study was one part of a therapy-optimization trial for the treatment of relapsed and refractory CNS-PNETs, pineoblastoma, or medulloblastoma. It consisted of three non-randomized arms: an intravenous chemotherapy arm (ivCHT arm) with carboplatin/etoposide, an oral chemotherapy arm (oCHT arm) with temozolomide, and a documentation arm (Doc arm) for patients who were not treated according to the aforementioned investigational study arms (Figure 1 and Figure 2).

Figure 1 shows the treatment algorithm at new diagnosis and in the P-HIT-REZ-Study: Following the new diagnosis, the patients were treated according to the guidelines of the German HIT-2000 protocol receiving tumor biopsy or resection, adjuvant chemotherapy and radiotherapy dependent on age, tumor residuum after primary tumor resection, and tumor spread. At relapse, all therapy modalities should be re-evaluated. Therapy selection of the different study arms in the P-HIT-REZ-Study was based on the recommendations of the treating physicians, dependent on preconditions of the patients (previous treatment, comorbidities, relapse pattern), and by the choice of the patients and their parents/guardians.

#### 2.1.3. Treatment Arms and Local Therapy

In the ivCHT arm, the patients received carboplatin and etoposide similar to the HIT-REZ 97 Study (carboplatin 200 mg/m^2^/d and etoposide 100 mg/m^2^/d, on d1–4, as 96-h continuous intravenous infusion, etoposide started 6 h after start of carboplatin, q21 to 28 days). In the oCHT arm, blockwise temozolomide (150 mg/m^2^/d, on d1–5, q21–28 days, the dose could be increased dependent on individual tolerance up to 200–250 mg/m^2^/d in subsequent cycles) was administered (Figure 2).

Based on the response to the phase II window study with intraventricular etoposide (response: at least stable disease or better) or on an individual decision by the treating physician, the patient could be treated in both chemotherapy arms with a simultaneous intraventricular therapy with etoposide via an Rickham/Ommaya Reservoir in an age-dependent dose (etoposide as Eto-Gry^®^ 20 mg/mL concentrate, GRY-Pharma GmbH, Kirchzarten, Germany, registration No. 45891.00.00, for application dilution of stock solution 1:100 with 0.9% sodium chloride, age dependent single dose: ≥3 months; 3 years <0.7 mg/d, ≥3 years; 30 years ≤ 1.0 mg/d, one single daily dose on d1–5).

Therapy response was measured after two and four therapy cycles in both treatment arms. If progression was detected, a switch to the opposing treatment arm was possible. If, after four cycles of therapy, a complete remission (CR) was achieved, patients additionally were eligible to receive high-dose chemotherapy (HDCHT) as a single course dependent on the previously given chemotherapy arm (HDCHT in ivCHT arm: carboplatin 500 mg/m^2^/d and etoposide 250 mg/m^2^/d, d-8 to d-5, as 96-h continuous intravenous infusion, etoposide started 6 h after start of carboplatin, thiotepa 150 mg/m^2^/d, d-8 to d-5, as one hour short infusion; HDCHT in oCHT arm: temozolomide 400 mg/m^2^/d, d-10 to d-6, orally as single dose, thiothepa 300 mg/m^2^/d, d-5 to d-3, as one hour short infusion; autologous peripheral blood stem cell transplantation (APBSCT) on day 0 in a dose of ≥2 × 10^6^ CD34 positive cells/kg body weight on day 0). Maintenance chemotherapy after HDCHT with either etoposide plus trofosfamide (etoposide 25 mg/m^2^/d and trofosfamide 100 mg/m^2^/d, d1–21, q28 days) in the ivCHT arm or continuation with temozolomide (equal dose as before mentioned) in the oCHT arm was recommended for a maximum of 24 months after the start of relapse chemotherapy or until progression.

If the response after four blocks was a partial response (PR) or stable disease (SD), maintenance therapy was recommended without HDCHT. In all cases of residual tumor lesion(s), after four blocks of relapse chemotherapy and a tumor response graded as PR, SD, or PD (progressive disease), local therapy should be re-evaluated by local centers in consultation with the trial office and the reference centers for neurosurgery and/or radiotherapy. If complete resection was expected to be achievable, tumor resection was recommended prior to HDCHT. In cases of previously non-irradiated patients and an age above 18 months, first irradiation at relapse was highly recommended, involving the whole craniospinal axis (CSA), the posterior fossa (PF), and, if present, the tumor bed of any metastasis. Concepts for dose and target volumes in these patients were based on the radiotherapy guidelines of the HIT’2000 Protocol (NCT00303810). In cases of previously irradiated patients (including irradiation of CSA, PF, and metastases, if present at new diagnosis), local re-irradiation at relapse was an option in case of one or two intracranial residual tumor lesion(s) or as local palliative irradiation for symptom control, e.g., in patients with an impending paraplegic syndrome.

#### 2.1.4. Assessment of Therapy Response

Overall response to the therapy was determined from volume change in measurable target lesions according to the McDonald criteria [15] (clear measurements possible in all three dimensions), presence of non-measurable non-target lesions, CSF cytology, and the occurrence of laminar meningeosis (Table 1). Radiological and pathological response assessments were conducted by central review for all patients.

Target lesions were assessed to have a CR if no residual was found in a follow-up MRI, a partial PR if the tumor volume was reduced by at least 50%, an SD if it decreased by less than 50% and increased by less than 25%, and a PD if it increased in volume by more than 25%. In non-target lesions, assessment of response could either be a CR of all lesions, a non-CR if any lesion could still be found, and PD if new lesions appeared or any lesion showed a clear progression. In CSF cytology, CR was defined as no detectable tumor cells (persistent or after previous detection), no response (NR) was defined as persistent detection of tumor cells, and PD as a new or recurring detection. Similarly, laminar meningeosis was assessed as CR if it was not detectable, NR if it persisted, and PD if it was found to be newly occurring or recurring. Objective response rate (ORR) was defined as the percentage of CR and PR of all evaluable patients in the ivCHT and oCHT arms. Best overall response was defined as the best response since initiation of systemic therapy in the ivCHT arm or oCHT arm until the next progression or recurrence and before any radiotherapy or tumor resection was performed at relapse.

#### 2.1.5. Local Therapy

The extent of resection was determined by contrast-enhanced lesion(s) in a T1-weighted MRI within 24 to 72 h post-operation. If no residual tumor and no contrast enhancement were found, the extent of resection was graded as a gross-total resection (GTR). If contrast enhancement in T1-weighted MRIs or non-enhancing changes in T2-weighted (T2) and T2 Fluid Attenuated Inversion Recovery (FLAIR) were found at the edge of the resection area, the extent was determined to be a near-total resection (NTR). Subtotal resections (STR) showed a reduction in tumor volume of at least 10% but less than 90%. If no or less than a 10% change in tumor volume was found, the resection was judged as a biopsy only. Tumor material was investigated by the local neuropathologist and centrally by the reference neuropathologist. Determination of molecular subgroup and further molecular genetic analyses were performed retrospectively, if stored tumor material were available. In the case of detection of a secondary malignancy, patients were excluded from the study analysis. Radiotherapy was evaluated according to total target doses and target volumes (covering CSI with boost or focal irradiation only).

#### 2.1.6. Safety Analysis

All patients who had received at least one cycle of systemic chemotherapy in the ivCHT arm or oCHT arm or at least one day of the first cycle with discontinuation due to toxicity reasons were evaluated for chemotherapy-associated toxicity. Adverse events were assessed according to the Common Terminology Criteria for Adverse Events of the National Cancer Institute (CTCAE version 3.0, https://ctep.cancer.gov/protocoldevelopment/electronic_applications/docs/ctcaev3.pdf, publish date: 9 August 2006, accessed on 10 November 2021).

### 2.2. Statistical Analysis

The study population was defined as per protocol population (PPP) and included all study patients who had received at least two cycles of systemic chemotherapy with at least 50% of the planned dose in the ivCHT arm or oCHT arm, or at least one cycle before discontinuation due to clinical or radiological rapid progression. PPPs were analyzed for efficacy endpoints as the best objective response rate, PFS, and OS from therapy starting in both chemotherapy arms, as well as for safety. The response and safety analysis were conducted as an explorative analysis.

All survival analyses were performed using the Kaplan–Meier method and estimated either post-relapse overall survival (OS_1stRD_) from the time of diagnosis of first recurrence/progression to death, or progression-free survival (PFS_1stRD_) from the diagnosis of first recurrence/progression to either further recurrence/progression or death. In order to compare the different chemotherapy arms, including first and multiple relapses, the survival analysis was also performed from the start of systemic relapse chemotherapy (PFS_TS_ and OS_TS_). Patients were censored if no event occurred by the last follow up. If not otherwise specified, the survival estimates are given as a median with its corresponding 95% confidence-interval (CI). To compare survival distributions between two or more groups, the log rank test was used and an alpha-value of 0.05 was chosen to test for statistical significance. Univariate and multivariate Cox regressions were used to approximate the effects of covariates on PFS_1stRD_ and OS_1stRD_. Their effect is given as a Hazard Ratio (HR). The Wald test was used to test for statistical significance, with an alpha-value of 0.05 chosen to reject the null hypothesis. To test for statistical significance between the rates of (objective) responses to different chemotherapies, Fisher’s exact test was used and an alpha-value of 0.05 was chosen to reject the null hypothesis.

Descriptive statistics and survival estimates were done using SPSS Version 27 (IBM Corp. Released 2020. IBM SPSS Statistics for Windows, Version 27.0. Armonk, NY: IBM Corp, USA)). Cox regressions were done using the survival package within R Version 4.0.3 (R Core Team (2020), R: A language and environment for statistical computing. R Foundation for Statistical Computing. Vienna, Austria. URL https://www.R-project.org/ accessed on 10 November 2021), while all graphics were compiled using the ggplot2 package.

### 2.3. Ethical Approval

All procedures in this study involving human participants were in accordance with the ethical standards of the institutional and national committees. The trial was conducted in accordance with the 1964 Helsinki Declaration and its later amendments or comparable ethical standards. The institutional review boards or ethics committees of all participating centers reviewed and approved the protocol. All parents/guardians and patients, where appropriate, gave their written informed consent for data collection and analysis.

## 3. Results

### 3.1. Clinical Characteristics

A total of 119 patients with recurrent CNS tumors were recruited onto the P-HIT-REZ 2005 trial between 2006 and 2013, of which 98 had a primary histological diagnosis of medulloblastoma. Three patients were subsequently excluded due to divergent histologic diagnoses (all high-grade gliomas) in further reference neuropathology analyses. Two further patients were excluded because of insufficient data, leaving 93 patients within this analysis (Figure 3). Median follow-up time, from the diagnosis of the first recurrence/progression, was 18.5 months (IQR: 8.3–42.3) at database lock on 31 December 2019.

Table 2 shows the patient characteristics of the patient cohort at first recurrence as well as of the study cohort at study entry. Patients were predominantly male (72.0%) and had a median age of 10.1 years (IQR: 6.9–16.1, range: 2.6–30.6) at the time of first recurrence, with a median time to recurrence from initial diagnosis of 23.6 months (IQR: 16.0–41.9). Histologically, tumors showing classical histology were most abundant in 81.7% of cases. Furthermore, 14.0% had desmoplastic, 3.2% had anaplastic, and 1.1% had large cell histology. Molecular data for subgrouping were available for 45.2% of patients within our cohort. Of these, 2.2% fell within the WNT, 3.2% within the SHH (TP53 mutation-status unknown), and 17.2% as well as 22.6% within subgroups 3 and 4, respectively. *Myc* and *MycN* amplification status was known in 15 patients with a detectable *Myc* amplification in 3 patients (2 patients in subgroup 3, 1 patient without a known subgroup) and without a detectable *Myc/MycN* amplification in 12 patients (5 patients each in subgroup 3 and 4, 2 patients in the SHH subgroup). Metastatic disease was present in 83 of the 93 recurrences (89.2%). Metastases most often affected the spinal cord, with two thirds of metastatic relapses occurring in an M3 stage. The study cohort was not too different from the cohort at first recurrence. Only one patient of the study cohort enrolled in the study at his second relapse; all others enrolled at their first recurrence/progression.

### 3.2. Treatment

The chemotherapy applied at first recurrence was dependent on the patients’ respective study-arm within the trial. Oral chemotherapy with temozolomide (oCHT arm) was the most commonly applied study treatment in 47 patients (50.5%, in 46 patients at 1st recurrence, in 1 patient at 2nd relapse), with a median of 4 cycles (range: 1 to 26 cycles). Intravenous chemotherapy with a combination of carboplatin and etoposide (ivCHT arm) was applied in 28 patients (30.1%) at first relapse with a median of 4 cycles (range: 2 to 8 cycles). Eleven patients (11.8%) received maintenance therapy with etoposide and trofosfamide after intravenous chemotherapy.

A total of 32 patients (34.4%) received, at first relapse and prior to any oral or intravenous chemotherapy, the phase II window trial with intraventricular etoposide. Three patients (5.9%) received phase II window therapy at further relapses.

Patients included in the documentation arm received other lines or schedules of systemic or local therapy, e.g., re-resection, intraventricular liposomal cytarabine, re-irradiation, and chemotherapy according to the decision of the treating physician, the patients, or their guardians.

Intraventricular chemotherapy was applied in 42 patients (45.2%) at first relapse and in 32 patients (34.4%) in the form of the phase II window trial as described above. A total of 18 patients (19.4%) received intraventricular etoposide beyond the phase II window study, 5 (5.4%) obtained liposomal cytarabine, and 3 (3.2%) received methotrexate.

A total of 18.3% of all patients (*n* = 17) were treated with high-dose chemotherapy at first relapse consisting of thiotepa/carboplatin/etoposide (*n* = 13), temozolomide/thiotepa (*n* = 3), or other agents (*n* = 1).

At first recurrence, 22 out of 93 patients (23.7%) received the tumor-volume reducing surgery (GTR, NTR, STR). Twenty patients (21.5%) received radiotherapy (RT), 11 (11.8%) of them as a re-irradiation (2nd RT) and 9 of them without any prior RT (1st RT), respectively (Table 3). Out of all 93 patients, 81 patients (87.1%) received radiotherapy during initial treatment, including CSI in 88.9% of these patients. All nine patients with a 1st RT at 1st recurrence received CSI. The median time interval from 1st to 2nd RT in the re-irradiated cohort was 20.7 months (range: 3.0–36.6). The median dose of irradiation at first relapse given to the tumor bed was 49.6 Gy (range: 30.0 to 60.0 Gy), the median dose to the posterior fossa was 55.0 Gy (range: 54.6 to 56.6 Gy), and to the craniospinal axis was 35.2 Gy (range: 24.0 to 44.2 Gy), respectively.

### 3.3. Response to Chemotherapy

The response assessment after two cycles and four cycles of systemic chemotherapy and the best overall response are shown in Table 4. The ORR in the ivCHT arm with carboplatin/etoposide with 51.8% after 2 cycles was significantly higher than in the oCHT arm with temozolomide with 18.2% (Fisher’s exact test: *p* = 0.015). The difference after four cycles was not significant (*p* = 0.086), although the number of patients with progression after four cycles was also higher in the oCHT arm. Regarding the best overall response rate ever achieved with systemic chemotherapy, it was significantly higher in the ivCHT arm than in the oCHT arm (Fisher’s exact test: *p* = 0.023). Regarding patients with available molecular subgroup information, there was no detectable significant difference in best ORR between subgroup 3 (*n* = 4/11, 36.4%) and subgroup 4 ((*n* = 6/18, 33.3%); *p* = 0.776).

### 3.4. Survival Dependent on Patient and Relapse Characteristics

#### 3.4.1. Survival in the Whole Cohort

**Figure 4:**
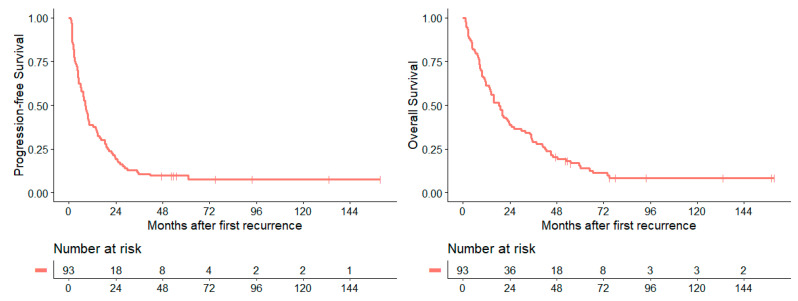
Survival curves for PFS_1stRD_ and OS_1stRD_ after diagnosis of first recurrence of medulloblastoma.

In the whole cohort of patients, median PFS_1stRD_ after diagnosis of first recurrence was 7.9 months (CI: 5.7–10.0), while median OS_1stRD_ was 18.5 months (CI: 13.6–23.5). Figure 4 shows the survival curves of the entire cohort.

#### 3.4.2. Survival Dependent on Sex

Male patients showed a significantly worse median PFS_1stRD_, with 7.2 months (CI: 5.3–9.1) compared to 9.6 months (CI: 1.8–17.4) in female patients (*p* = 0.022). The median OS_1stRD_ was 18.4 months (CI: 12.4–24.3) in male patients and 20.1 months (CI: 9.9–30.3) in female patients (*p* = 0.160).

#### 3.4.3. Survival Dependent on Histological Entities

Patients with a large-cell or anaplastic histology showed a significantly worse median PFS_1stRD_ (0.9 months (CI: 0.0–1.9)) and OS_1stRD_ (1.6 months (CI: 0.0–3.9)) compared to all other histological types (*p* < 0.001 for both PFS_1stRD_ and OS_1stRD_ compared to classical type; *p* = 0.001 both PFS_1stRD_ and OS_1stRD_ compared to desmoplastic/nodular type). No statistically significant differences in survival were found between classic and desmoplastic/nodular histology. The median PFS_1stRD_ was 8.1 months (CI: 6.4–9.7) in patients with tumors of classic histology, compared to 9.4 months ((CI: 3.0–15.9), *p* = 0.328) in patients with desmoplastic histology, while the medians of OS_1stRD_ were 18.5 months (CI: 13.8–23.3) and 26.0 months ((CI: 3.8–48.2), *p* = 0.468), respectively.

#### 3.4.4. Survival Dependent on Molecular Subgroup

As specified in Table 5, our analysis showed the best survival in group 4 medulloblastoma with a median PFS_1stRD_ of 7.2 months (CI: 4.9–9.5) and OS_1stRD_ of 20 months (CI: 15.1–24.9) compared to other molecular subgroups. Group 3 medulloblastomas (*Myc/MycN* status known in 7/16 patients, 2/7 with *Myc* amplification) was associated with a median PFS_1stRD_ of 4.9 months (CI: 3.2–6.0) and median OS_1stRD_ of 9.8 months (CI: 8.2–11.3). Within molecular subgroup SHH (TP53 mutation-status unknown) a median PFS_1stRD_ of 2.4 months (CI: 0–5.4) and a median OS_1stRD_ of 2.4 months (CI: 0.4–4.4) were found as well. None of the analyses were associated with a significant p-value, considering the small number of patients, except for the comparison of OS_1stRD_ between group 3 and 4 (*p* = 0.014).

#### 3.4.5. Survival Depending on Disease Stage at 1st Recurrence/Progression

Isolated local relapses (*n* = 10) showed a tendency to an improved survival median PFS_1stRD_ of 14.7 months (CI: 0.0–38.8, *p* = 0.191), a median OS_1stRD_ of 48.3 months (CI: 32.6–63.9, *p* = 0.027) compared to distant-only relapses. No differences were found for distant-only relapses (*n* = 61) compared to patients with combined relapses (*n* = 22). Distant-only relapses showed a median PFS_1stRD_ of 8.2 months (CI: 6.5–9.9) and OS_1stRD_ of 15.8 months (CI: 10.8–20.8), compared to a median PFS_1stRD_ of 6.5 months ((CI: 2.5–10.5); *p* = 0.757) and OS_1stRD_ of 15.6 months ((CI: 1.3–30.0); *p* = 0.850) in combined relapses.

Comparing the survival of patients with M2 stage at first recurrence (*n* = 25) to the large group of patients with an M3 stage (*n* = 55), we also found a not significantly improved median PFS_1stRD_ for the M2 group (9.9 months (CI: 6.2–13.5)) over the M3 group (5.8 months (CI: 3.6–9.0), *p* = 0.073), but a significantly improved OS_1stRD_ (M2: 29.9 months (CI: 7.6–52.1), M3: 14.0 months (CI: 9.2–18.8), *p* = 0.029).

#### 3.4.6. Survival Depending on Time to 1st Recurrence/Progression

**Figure 5:**
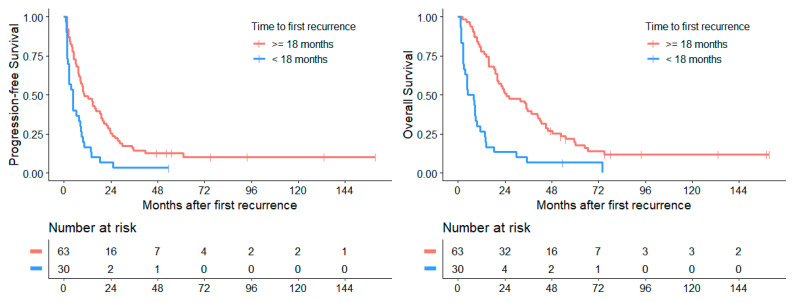
Time to recurrence from initial diagnosis of <18 months correlates with a significantly worse PFS_1stRD_ (*p* < 0.001) and OS_1stRD_ (*p* < 0.001).

Patients with a time of under 18 months to their first relapse after initial diagnosis (*n* = 30) exhibited a significantly worse median PFS_1stRD_ of 3.2 months (CI: 1.3–5.1) and a median OS_1stRD_ of 4.9 months (CI: 0.0–10.0) after first recurrence than patients with a longer progression-free interval (*n* = 63), who after first relapse showed a median PFS_1stRD_ of 10.1 months (CI: 5.1–14.6; *p* < 0.001) and median OS_1stRD_ of 24.8 months (CI: 11.7–38.0; *p* < 0.001, Figure 5). The 2 subgroups at both sides of the 18 months cut off showed significant differences in their distribution of group 3 and 4 subtypes (Fisher’s exact test: *p* = 0.003). Within the group of patients that relapsed early, group 3 subtypes were more abundant than group 4 (9 vs. 2, respectively), while in later relapses group 4 far outnumbered group 3 subtypes (19 vs. 7, respectively). Recurrences after more than five years (range: 5.1 to 18.4 years) after initial diagnosis were found in 11 patients (11.8%).

### 3.5. Survival Dependent on Treatment Modalities

#### 3.5.1. Chemotherapy

Analyzing the two most common initial systemic chemotherapies at first relapse, a survival advantage for the ivCHT with carboplatin and etoposide (median PFS_TS_ of 8.8 (CI: 3.7–13.8), median OS_TS_ of 27.6 months (CI: 9.1–46.1)) was found in comparison to oCHT with temozolomide (median PFS_TS_ of 4.0 months (CI: 1.4–6.7), median OS_TS_ of 14.1 months (CI: 7.2–21.0); *p* = 0.025 for PFS_TS_ and *p* = 0.070 for OS_TS_).

An objective response (CR/PR) to either initial chemotherapy at first relapse showed an improved median PFS_TS_ and OS_TS_. Median PFS_TS_ after a CR or PR was 19.9 months (CI: 15.2–24.6), compared to only 3.7 months (CI: 2.3–5.1) if no objective response was achieved (*p* < 0.001). Median OS_TS_ was also improved at 34.6 months (CI: 0–75.5) compared to 11.5 months ((CI: 6.0–17.1); *p* = 0.006).

Intraventricular therapy at first relapse was applied most often in the form of the phase II window trial (*n* = 32) treated with intraventricular etoposide. Patients who went through the phase II window trial showed a PFS_1stRD_ of 8.3 months (CI: 5.5–11.2) and an OS_1stRD_ of 14.4 months (CI: 7.2–21.6).

Intraventricular chemotherapy at first relapse (*n* = 43) in general was not related to significant PFS_TS_ (*p* = 0.170) or OS_TS_ benefits (*p* = 0.274). Patients with intraventricular chemotherapy had a median PFS_TS_ of about 7.3 months (CI: 5.0–9.5) and a median OS_TS_ of 17.6 months (CI: 10.8–24.4), patients without this treatment approach had a median PFS_TS_ of 4.0 months (CI: 0–8.0) and a median OS_TS_ of 15.8 months (CI: 6.9–24.1).

**Figure 6:**
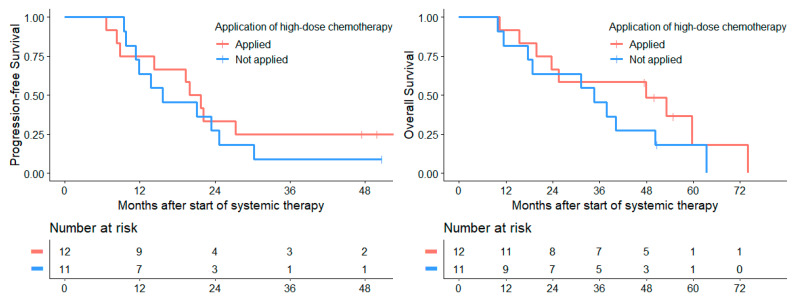
Application of high-dose chemotherapy after CR/PR at MRI after 4 cycles of initial systemic chemotherapy did not improve either PFS_TS_ (*p* = 0.51) or OS_TS_ (*p* = 0.25).

Application of high-dose chemotherapy at first relapse in patients with a CR/PR prior to high-dose therapy (*n* = 12) was associated with no significant benefits for median PFS_TS_ (19.9 months (CI: 15.9–24.0)) compared to patients with a CR/PR after 4 cycles of chemotherapy without an application of high-dose therapy (*n* = 11; median PFS_TS_ of 15.7 months (CI: 5.7–25.6); *p* = 0.509). The median OS_TS_ in cases of high-dose therapy was 47.8 months (CI: 11.1–84.6), compared to 34.6 months ((CI: 14.0–55.2); *p* = 0.253) in patients without this therapy form (Figure 6).

#### 3.5.2. Surgery

**Figure 7:**
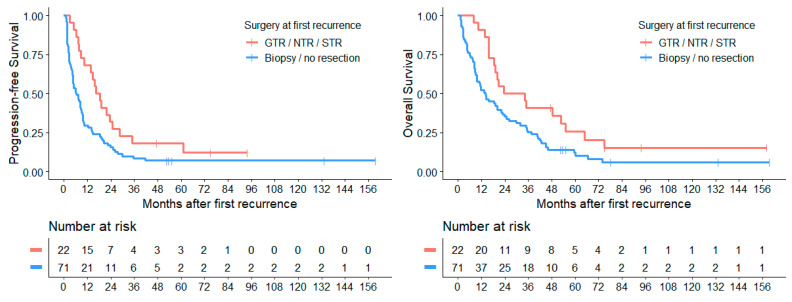
Surgery at first recurrence significantly improves both PFS_1stRD_ (*p* = 0.015) and OS_1stRD_ (*p* = 0.025).

Comparing the survival of patients with or without tumor volume reducing surgery (Figure 7) we found a significantly (*p* = 0.008) improved median PFS_1stRD_ in patients who received non-biopsy surgery (GTR, NTR, STR) with 15.8 months (CI: 11.6–20.0) over those who received no debulking surgery with 6.0 months (CI: 3.2–8.8). Median OS_1stRD_ was also improved when debulking surgery was performed (23.4 months (CI: 6.5–40.3) vs. 14.0 months (CI: 7.0–20.9), *p* = 0.025).

Between different extents of resection, no significant difference was found in median PFS_1stRD_ (*p*_GTR_ vs. _NTR_ = 0.767; *p*_GTR_ vs. _STR_ = 0.541; *p*_NTR_ vs. _STR_ = 0.888) as well as in median OS_1stRD_ (*p*_GTR_ vs. _NTR_ = 0.957; *p*_GTR_ vs. _STR_ = 0.622; *p*_NTR_ vs. _STR_ = 0.69). When GTR was achieved, patients had a median PFS_1stRD_ of 14.6 months (CI: 9.6–19.7) and OS_1stRD_ of 34.1 months (CI: 9.2–59.0). NTR was associated with a median PFS_1stRD_ of 10.4 months (CI: 2.7–18.1) and OS_1stRD_ of 20.6 months (CI: 7.7–33.5) and STR with a median PFS_1stRD_ of 16.5 months (CI: NA–NA) and OS_1stRD_ of 23.4 months (CI: 15.3–31.5).

#### 3.5.3. Radiotherapy

The application of radiotherapy at first relapse as 1st or 2nd RT showed a significant survival benefit with a median PFS_1stRD_ of 16.5 months (CI: 0–40.7), compared to only 7.2 months (CI: 4.4–10.0) when it was not applied (*p* = 0.001). Median OS_1stRD_ was 34.8 months (CI: 0–72.0) in contrast to a time of 15.8 months (CI: 9.7–21.8) when no radiotherapy was applied (*p* = 0.016). Four patients (4.3%) received local irradiation only, while 13 patients (14.0%) received CSI with or without boost. In three patients (3.2%), no specific information was given on the target volume. Patients with local irradiation only showed a median PFS_1stRD_ of 8.3 months (CI: 5.7–11.0), while patients who received CSI had a median PFS_1stRD_ of 25.2 months (CI: 1.9–48.4), which was not statistically significant (*p* = 0.186). Median OS_1stRD_ with local irradiation was 8.3 months (CI: 0–17.8) and 35.4 months (CI: 0–96.4) with CSI (*p* = 0.139), respectively.

**Figure 8:**
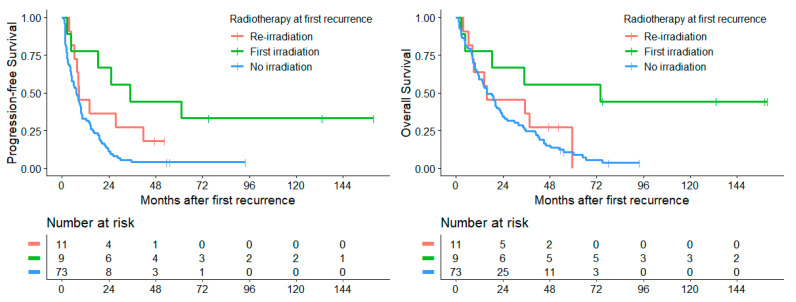
In radiotherapy-naïve patients, radiotherapy significantly improves both PFS_1stRD_ (*p* = 0.004) and OS_1stRD_ (*p* = 0.005) compared to patients with either re-irradiation or no radiotherapy at first recurrence. Re-irradiation showed no significant increase in PFS_1stRD_ (*p* = 0.078) and OS_1stRD_ (*p* = 0.54) compared to patients without radiotherapy. Improvements in survival between first-irradiated patients and re-irradiated patients did not reach significance for PFS_1stRD_ (*p* = 0.21) and OS_1stRD_ (*p* = 0.13).

11 patients (11.8%) were irradiated both within primary and relapse therapy (2nd RT) and showed a median PFS_1stRD_ of 8.7 months (CI: 1.7–15.8) and a median OS_1stRD_ of 15.8 months (CI: 0–44.3). Comparing this group of re-irradiated patients with patients without radiotherapy, we did not find a significant increase in PFS_1stRD_ (*p* = 0.078) and OS_1stRD_ (*p* = 0.540). The first RT at first relapse (*n* = 9) resulted in a median PFS_1stRD_ of 33.9 months (CI: 8.4–59.3) and OS_1stRD_ of 73.8 months (CI: 0–187.6, Figure 8).

The above-mentioned 11 patients with 2nd RT showed a PFS from the end of 1st RT to first relapse of 14.8 months (CI: 10.5–19.1). In contrast, median PFS from the end of 2nd RT to 2nd relapse or death in this group was 2.8 months (CI: 2.7–2.9).

The patient group receiving 1st RT at relapse had a median age of 2.9 years (IQR: 2.7–3.9) at initial diagnosis and 5.3 years (IQR: 4.3–7.5) at 1st relapse. Within this group, four patients received surgery, which did not significantly impact PFS or OS (*p* = 0.92 and *p* = 0.71, respectively). All patients received chemotherapy (3 oCHT, 4 ivCHT, 1 HIT-SKK and 1 vincristine parallel to radiotherapy).

Four patients received radiotherapy after GTR/NTR. For these patients, no improvement in median PFS_1stRD_ and OS_1stRD_ was found for adjuvant radiotherapy when compared to patients without adjuvant RT (*n* = 15) (median PFS_1stRD_ 33.9 months (CI: NA–NA) vs. 12.8 months (CI: 6.8–18.8), *p* = 0.052; median OS_1stRD_ 34.8 months (CI: 4.4–65.3) vs. 20.6 months (CI: 1.8–39.5), *p* = 0.255). If, however, no or only incomplete surgery was performed prior to irradiation, a markedly improved median PFS_1stRD_ (14.1 months (CI: 0–30.0) vs. 4.8 months (CI: 2.2–7.3), *p* = 0.003) and median OS_1stRD_ (18.4 months (CI: 0–59.5) vs. 13.2 months (CI: 7.6–18.8), *p* = 0.033) were found for adjuvant RT.

### 3.6. Survival Rates

Additionally, to the above-mentioned survival analysis, Table 6 applies an overview about the 2-, 5-, and 10-year PFS_1stRD_ and OS_1stRD_ rates of the whole cohort at first recurrence/progression and the 2-, 5-, and 10-year PFS_1stRD_ and OS_1stRD_ rates dependent on the different patient, disease, and treatment characteristics. These results demonstrate rapid decreasing in survival within the first two years following diagnosis of first recurrence/progression of medulloblastoma with very low survival rates after five and ten years.

### 3.7. Cox Regression Analysis

For our Cox regression analyses, we used gender, metastases at first recurrence, desmoplastic histology (opposed to classic or anaplastic histology), age at first recurrence (under 6 and over 16 years), time to first recurrence from initial diagnosis (<vs. ≥18 months), extent of resection (GTR/NTR/STR vs. biopsy/no resection), application of radiotherapy, and response to chemotherapy at first recurrence (CR/PR vs. SD/PD) as well as age under 6 years or over 16 years as covariates. Due to the high number of missing data, molecular subgroups were not included within this analysis.

Table 7 shows the results of both the univariate and multivariate analysis. Application of radiotherapy and an objective response to chemotherapy at first recurrence improved both PFS and OS significantly in univariate and multivariate regression. A time to first recurrence of <18 months was a strong predictor for a worse PFS and OS in both analyses. Female gender and non-biopsy surgery improved PFS in univariate analysis but did not alter survival significantly within the multivariate analysis. In multivariate analysis, an age under six years showed improved OS.

### 3.8. Toxicity Analysis

#### 3.8.1. Toxicity of Conventional Chemotherapy Arms

Toxicity analysis was carried out for each of the first four cycles in the ivCHT and oCHT arm (Table 8). As expected, the ivCHT arm was clearly associated with a significant higher rate of hematological toxicity, intestinal mucositis, and infections/febrile neutropenia (comparison the rate of CTC grade 3 and 4 in both arms: *p* < 0.001). There was no therapy-related mortality. Even though the rate of infections in the oCHT arm was low, there were documented varicella zoster infections in one patient and another severe co-infection with a pneumocystis jirovecii pneumonia and invasive aspergillosis in another patient being complicated by respiratory distress and multiple subcutaneous abscesses. Ototoxicity was reported in both arms probably as cumulative toxicity to first-line and relapse treatment with platin-containing agents and radiotherapy. As causes for the reported neurotoxicity (symptoms of increased intracranial pressure and seizures), the leptomeningeal disease manifestation also has to be assumed as an additional potential risk factor for its occurrence in most cases. There was no increase of toxicity from cycle to cycle in both chemotherapy arms.

Toxicity of simultaneous intraventricular therapy with etoposide in the ivCHT and oCHT arm could not clearly be differentiated from the toxicity associated with the systemic therapy and therefore was not depicted separately in Table 8. Adverse events probably or definitively related to the concurrent intraventricular therapy were only reported in 2 patients treated in the oCHT arm: 1 event in a 10-year-old patient whose treatment was complicated by a febrile infection of the Ommaya reservoir caused by Staphylococcus hominis requiring reservoir explantation and intravenous antibiotic therapy, and another event in a 28-year-old patient with an accidental overdosing of intraventricular etoposide (9 mg instead of planned 1mg dose on day 1 of the first therapy cycle) causing a mild transient headache.

#### 3.8.2. Toxicity of High-Dose Chemotherapy

Due to the small number of patients (*n* = 17) who were treated with high-dose chemotherapy, the toxicity analysis for this modality were performed together for all different used high-dose regiments (Table 9). As expected, severe hematological toxicity was documented in all patients. No therapy-related death occurred. Associated with the therapy-induced severe leukopenia and granulocytopenia and severe oral and intestinal mucositis, in the majority of patients severe infections and febrile neutropenia were observed. One life-threatening infection was caused by an invasive adenovirus infection and was complicated by intestinal hemorrhage, septic shock, and transient acute renal failure. Other relevant non-hematological toxicities were transient neurotoxicity, transient skin and hepatotoxicity, and permanent ototoxicity. Comparing the mostly used HDCHT regiments (thiotepa/carboplatin/etoposide, *n* = 13, vs. temozolomide/thiotepa, *n* = 3) no differences in severity of reported adverse events were observed.

#### 3.8.3. Long-Term Toxicity

Long-term sequelae were reported in 35 of 93 patients (37.6%), where an underreporting bias cannot be excluded due to disease-related death in the majority of patients within less than two years after first recurrence/progression and missing evaluation especially in palliative patients. Ototoxicity was the most relevant toxicity notified in 15 patients (CTC°2 in 9 patients, CTC°3 in 5 patients needing bilateral hearing aids). Additionally, partial pituitary insufficiency (mostly as hypothyroidism and growth hormone deficiency) was documented in 16 patients, cardiotoxicity of CTC°2/°3 in 2 patients, disturbances of the memory and concentration in 3 patients, and secondary malignancies in 2 patients (one fibro-histiocytic tumor of the petrous bone, one melanoma of the foot).

## 4. Discussion

The presented cohort includes 93 evaluable patients with refractory or recurrent medulloblastomas treated with various treatment regimens within the German P-HIT-REZ 2005 Study. We investigated clinical and treatment characteristics and their impact on patients’ short- and long-term survival as well as on the safety of study therapy.

In our cohort, patients were in median 10.1 years old, mainly between 6 and 16 years (54.9%), with a median time to recurrence after initial diagnosis of 23.6 months. As other studies have already shown, male patients (72.0%) also lead in our analyses, as does classical histology (81.7%) and metastatic disease at the time of first relapse diagnosis (89.3%) [1,16,17]. The main molecular subtypes at relapse were groups 3 and 4, being comparable with other studies, although data on molecular biology could not be obtained in more than half of the patients due to a lack of stored tumor tissue [1,17,18].

Our overall survival analyses of the entire cohort revealed similar short-term and long-term results to those from previous published multicenter studies of relapsed medulloblastomas, inclusively the HIT-REZ 97 Study, with an OS_1stRD_ rate after 2, 5, and 10 years of 38.7%, 15.5%, and 8.4%, respectively [1,3,11,16,19,20]. The best short-term results so far with a 2-year OS of 68.6% were reported by Peyrl et al. (2012) in a monocenter pilot study with an antiangiogenic multi-agent regimen (intravenous bevacizumab, oral thalidomide, celecoxib, fenofibrate, etoposide, cyclophosphamide, intraventricular etoposide, and liposomal cytarabine) [8]. Results of the subsequent multicenter phase II study using the same metronomic regimen (NCT01356290) are pending.

About one third of patients in our study suffered from an early recurrence (<18 months after initial tumor diagnosis) showing significantly worse survival (median PFS_1stRD_ of 3.2 months, median OS_1stRD_ of 4.9 months). In this cohort, the molecular subgroup 3 was mainly found to confirm previous published results [1,17,18].

Regarding local therapy, previous studies suggest a positive impact of surgery on survival rates at medulloblastoma relapse [1,2,9,19]. Our study analyses have also shown that patients who underwent tumor debulking surgery (GTR, NTR, or STR) have a significant improved median PFS_1stRD_ and OS_1stRD_ in comparison to those who received no surgery or a biopsy only. Between different extents of resection (GTR, NTR, or STR), we could not find any significant difference, probably due to the disseminated disease being present at relapse diagnosis in about 90% of patients and the small number of patients in each subgroup. Regarding the poor prognosis of recurrent disease, a tumor biopsy at relapse seems to be justified to investigate the histology and molecular biology of relapsed tumor lesion(s). At recurrence, the molecular subtype in a given subgroup may differ from the initial diagnosis and could show intratumorally or spatially heterogeneity due to subclonal evolution or newly occurring genetic alterations. In contrast, the histological and molecular groups typically remain stable between new diagnosis and relapse as well as between primary tumor and metastases [17,21,22,23,24,25,26]. Detection of genetic alterations, such as *TP53, SMO, PTCH* mutations and *MYC/MYCN* amplification or other specific alterations, might be helpful to identify actionable targets for future therapies and reasonable combination therapies. In addition, second malignancies, such as radiotherapy-induced glioblastomas, should be excluded [27,28,29]. Based on our own and the published data, a re-biopsy or tumor resection at relapse is strongly recommended in order to investigate tumor histology and biology for further therapy decisions.

Application of radiotherapy at relapse showed a significant benefit in our survival analysis. This effect was attributable primarily to patients with first radiation at relapse. In contrast, there was no survival advantage for patients with re-radiation at first relapse in comparison to patients without re-irradiation. The multivariate Cox regression analysis confirmed these results with an HR of 0.15 and 0.17 for PFS and OS, respectively, in the cohort with first radiation at first recurrence. In contrast to other studies, we could not detect a significant positive impact of CSI in comparison to local irradiation [1,17,30]. However, we could show a tendency to a better PFS and OS in patients treated with CSI at relapse, which was based mainly on the cohort of patients with first radiotherapy at first relapse where all patients had received a CSI. Additionally, a significantly improved PFS and OS were found for irradiation in patients with residual tumors, i.e., where no resection, only biopsy or STR, were performed. Taken into account the overlap between the different analyzed groups, the small number of patients in each group and the different radiotherapeutic preload following risk-adapted first-line treatment further investigations of (re-)irradiation at relapse in a larger cohort are warranted in order to clarify its role for prognosis and long-term side effects [31]. In current first-line studies, as in the SIOP PNET5 MB Trial, reduced risk-adapted CSI doses in patients with low risk and standard risk medulloblastoma are used, very likely enabling re-irradiation with a second CSI in future cases of relapse [32]. In case of metastatic disease at newly diagnosed medulloblastoma in non-infant patients, the radiotherapeutic preload of the craniospinal axis will remain high with limited options for re-irradiation.

Chemotherapy with carboplatin and etoposide in the ivCHT arm turned out to be an adequate systemic chemotherapy with significantly improved best objective response rates and PFS/OS from therapy start in comparison to temozolomide monotherapy in the oCHT arm. The results of the ivCHT arm were similar to results of the HIT-REZ 97 Study and improved in comparison to other published, less intensive regimens, such as TOTEM (temozolomide and topotecan) or TEMIRI (temozolomide and irinotecan ± bevacizumab) [3,11,20,33]. An objective response (CR/PR) to either initial chemotherapy at first relapse can serve as a surrogate parameter for in vivo chemotherapy sensitivity and was associated with a significantly improved PFS and OS.

No significant survival benefit was found through the administration of intraventricular therapy, although small patient numbers could influence these results. It is known that intraventricular chemotherapy acts only as local therapy for the treatment of floating tumor cells in the CSF and of deposits on the leptomeninges and pachymeninges. However, mostly used agents are unable to penetrate the meningeal surface further than a few cell layers and to cross the intact CSF-brain barrier in a relevant amount [34,35,36]. Therefore, intraventricular therapy is unsuitable to treat parenchymatous tumor lesions efficiently and additional local and systemic treatment modalities are necessary in these cases.

At the end of the last century, HDCHT with APBSCT has been widely used in the consolidation treatment of recurrent medulloblastomas. Lessons we have learned from these non-randomized trials are that patients with minimal residual disease burden and/or local relapses with an option for local re-irradiation and infants suffering from local relapses might have individual survival benefits from this approach. However, in the majority of pre-irradiated patients with diffusely disseminated diseases at recurrence, long-term survival remains poor and therapy-related morbidity with HDCHT is high and sometimes life-threatening [7,10,37,38,39]. In this study, as in our previous HIT-REZ 97 Study, we could demonstrate that in chemotherapy-responsive recurrent disease by utilizing all local-therapeutic options the HDCHT does not lead to an overall survival benefit and is no longer recommended [11].

Regarding the therapy related toxicity in our study arms, we observed the expected higher hematological and non-hematological, but manageable toxicity with the intensive carboplatin/etoposide therapy in the ivCHT arm. Temozolomide monotherapy in the oCHT arm were mostly associated with mild and moderate adverse events. However, severe opportunistic fungal and viral infections were observed in individual patients most likely associated with therapy-related severe lymphopenia. The observed toxicity with the high-dose and conventional chemotherapy was comparable with similar high-dose, intensive or less intensive regiments, in comparison to previous published studies without therapy-related deaths [3,7,11,20,33,40].

Based on the recent available knowledge in 2005 the treatment regimens in the P-HIT-REZ 2005 Study did not consider the histological and molecular MB entities for therapy stratification, although these have been shown to represent biologically and clinically different disease entities [5,27]. Additionally, due to the non-randomized study design, the small number of patients in investigated subgroups, the different preload of chemotherapy and radiotherapy in patients, the high rate of missing information about biological tumor characteristics and the neurocognitive outcome in long-term survivors, the results of our study must be considered limited. Even when initial chemotherapy was precisely prescribed in the oral and intravenous arms, further therapy was chosen by the responsible physicians after progression. This makes comparability difficult; in addition, treatment success is affected by bias due to individual decision making. For example, more intensive therapies could have been chosen for those with expected better long-term outcomes, while less intensive oral therapy was chosen in a more palliative setting. These potential effects should be reduced in our analyses by analyzing the first relapse alone, as patients should still have received homogeneous therapy following the two chemotherapy arms. In addition, bias develops due to the intention of a palliative or curative treatment plan, as diagnostic studies such as MRI were used more infrequently in palliative settings, which may confound PFS.

## 5. Conclusions

In summary, our study showed that the strongest predictor for worse survival was the time to first recurrence of less than 18 months. Tumor debulking surgery and (re-)irradiation might improve the patients’ survival. Nowadays, because RT at first diagnoses tends to be applied rather with reduced cranio-spinal doses and smaller local boost volumes, we may be able to administer 2nd RT to more patients safely, hopefully increasing efficacy of our treatment strategies in future. Intensive systemic chemotherapy with carboplatin and etoposide (ivCHT) showed a survival advantage with a 5-year OS_1stRD_ of 32.4% in comparison to temozolomide (oCHT) with 3.1%. No survival advantage was detected with the use of intraventricular therapy and HDCHT with APBSCT, despite survival benefits in individual patients, cannot be excluded.

Future studies in recurrent medulloblastomas should focus on investigating tumor biology, additionally with the asservation of tumor material allowing a comparison of primary and relapse tumors and to identify driver mutations and potential actionable targets for therapy stratification, enrollment in early clinical trials or an individual target-driven therapy, and exclusion of secondary malignancies. New approaches, such as targeted therapies, immunotherapy, antiangiogenic therapy, and reasonable combined therapy approaches, might be investigated with consideration of the different biology of defined MB entities [1,3,6,8,17]. It would be desirable to establish international registries in the future to recruit more patients in such studies, so that meta-analyses with high evidence can be performed and more specific therapy recommendations can be formulated. Overall, larger case-numbers are needed to draw significant conclusions on potential benefits for survival.

## Figures and Tables

**Table 1:** Determination of Overall response.

Target Lesion	Non-Target Lesions and/or Nodular Meningeosis	New Lesions	CSF Cytology	Laminar Meningeosis	“Overall Response”
CR	CR	No	CR	CR	CR
CR	Non-CR	No	CR	CR	PR
PR	CR/Non-CR	No	CR	CR	PR
SD	CR/Non-CR	No	CR/NR	CR/NR	SD
CR/PR/SD	CR/Non-CR	No	NR	CR/NR	SD
CR/PR/SD	CR/Non-CR	No	CR/NR	NR	SD
PD	All	Yes or No	All	All	PD
All	PD	Yes or No	All	All	PD
All	All	Yes	All	All	PD
All	All	Yes or No	PD	All	PD
All	All	Yes or No	All	PD	PD

**Table 2:** Clinical Characteristics at first recurrence and at study relapse.

Clinical Characteristics	Subgroup/ Value	Number of Patients at 1st Recurrence (%)	Numbers of Patients at Study Relapse (%)
Sex	male	67 (72.0)	67 (72.0)
	female	26 (28.0)	26 (28.0)
Number of relapses	refractory	20 (21.5)	20 (21.5)
	1st	73 (78.5)	72 (77.4)
	2nd	-	1 (1.1)
Age at diagnosis of 1st recurrence or at study relapse	median	10.1 years	10.1 years
	range	2.6–30.6 years	2.6–30.6 years
	IQR	6.9–16.1 years	6.9–16.1 years
	<6 years	19 (20.4)	19 (20.4)
	>16 years	23 (24.7)	23 (24.7)
Time to 1st recurrence after initial diagnosis	median	23.6 months	23.6 months
	range	1.3–220.2 months	1.3–220.2 months
	IQR	16.0–41.9 months	16.0–41.9 months
Chang stage at 1st recurrence or at study relapse	M0	10 (10.7)	10 (10.7)
	M1	1 (1.1)	1 (1.1)
	M2	25 (26.9)	25 (26.9)
	M3	55 (59.1)	55 (59.1)
	M4	2 (2.2)	2 (2.2)
Extent of relapse	localized only	10 (10.7)	10 (10.7)
	disseminated only	61 (65.6)	61 (65.6)
	combined	22 (23.7)	22 (23.7)
Histological subgroups	classic	76 (81.7)	76 (81.7)
	desmoplastic	13 (14.0)	13 (14.0)
	large cell/anaplastic	4 (4.3)	4 (4.3)
Molecular subgroups	group 4	21 (22.6)	21 (22.6)
	group 3	16 (17.2)	16 (17.2)
	SHH	3 (3.2)	3 (3.2)
	WNT	2 (2.2)	2 (2.2)
	no data	51 (54.8)	51 (54.8)

**Table 3:** Local and systemic treatment of patients at first relapse.

Treatment	Therapy Arms/ Therapy Characteristics	Therapy Subgroups	Number of Patients at First Relapse *n* = 93 (%)	Number of Patients at ≥2nd Relapse *n* = 51 (%)
Chemotherapy	all	yes	93 (100)	6 (11.8)
	oCHT arm (temozolomide)	all	46 (49.5)	1 (2.0)
		with prior phase II window	18 (19.3)	1 (2.0)
		with simultaneous ivc etoposide	6 (6.4)	1 (2.0)
		with shift to oral	6 (6.4)	0 (0)
		etoposide/trofosfamide		
		with shift to ivCHT	7 (7.5)	0 (0)
	ivCHT arm (carboplatin/ etoposide)	all	28 (30.1)	0 (0)
		with prior phase II window	7 (7.5)	0 (0)
		with simultaneous ivc etoposide	8 (8.6)	0 (0)
		with shift to oCHT	5 (5.4)	0 (0)
	doc arm	all	19 (20.4)	5 (9.8) ^1^/37 (72.5) ^2^
		systemic chemotherapy	13 (14.0)	5 (9.8) ^1^/37 (72.5) ^2^
		with prior phase II window	6 (6.4)	1 (2.0) ^1^/14 (27.5) ^2^
Intra-ventricular chemotherapy	all	yes	43 (46.2)	9 (17.6)
	agents	phase II window trial, etoposide	32 (34.4)	3 (5.9)
		simultaneous etoposide to	18 (19.4)	2 (3.9)
		systemic chemotherapy		
		liposomal cytarabine (doc arm)	5 (5.4)	5 (9.8)
		methotrexate (doc arm)	3 (3.2)	1 (2.0)
High-dose chemotherapy	all	yes	17 (18.3)	1 (2.0)
	agents	thiotepa/carboplatin/etoposide	13 (14.0)	0 (0)
		temozolomide/thiotepa	3 (3.2)	0 (0)
		other agents	1 (1.1)	1 (2.0)
Surgery	all	yes	22 (23.7)	11 (21.6)
	extent of resection	GTR	12 (12.9)	4 (7.8)
		NTR	7 (7.5)	2 (3.9)
		STR	3 (3.2)	0 (0)
		unknown	0 (0)	5 (9.8)
Radiotherapy	all	yes	20 (21.5)	16 (31.4)
	sequence	RT as 1st RT	9 (9.7)	1 (2.0)
		RT as 2nd RT	11 (11.8)	15 (29.4)
	target volume	CSI only	2 (2.2)	0 (0)
		CSI with boost	11 (11.8)	2 (3.9)
		local tumor bed only	4 (4.3)	9 (17.6)
		unknown	3 (3.2)	5 (9.8)

^1^ patients initially assigned to doc arm; ^2^ patients shifted to the doc arm following further progression/multiple relapses.

**Table 4:** Response to chemotherapy arms after two and four cycles and best overall response.

Study Arm and Time Point	CR *n* (%)	PR *n* (%)	SD *n* (%)	PD *n* (%)	DOD *n* (%)	n. e. *n*	ORR %	*p*-Value
ivCHT after 2 cycles	3 (11.1)	11 (40.7)	9 (33.3)	4 (14.8)	0 (0)	1	51.8	0.015
oCHT after 2 cycles	2 (4.5)	6 (13.6)	15 (34.1)	19 (43.2)	2 (4.5)	3	18.2	
ivCHT after 4 cycles *	6 (23.1)	7 (26.9)	6 (23.1)	7 (26.9)	0 (0)	2	50.0	0.086
oCHT after 4 cycles *	2 (4.3)	9 (19.6)	12 (26.1)	20 (43.5)	3 (6.5)	1	23.9	
ivCHT Best overall response	6 (22.2)	12 (44.4)	7 (25.9)	2 (7.4)	0 (0)	1	66.7	0.023
oCHT Best overall response	3 (6.5)	13 (28.3)	14 (30.4)	15 (32.6)	2 (4.3)	1	34.8	

CR: complete response; PR: partial response; SD: stable disease; PD: progressive disease; DOD: death of disease; n.e.: not evaluable; ORR: objective response rate (defined as CR+PR/all evaluable patients). * To avoid a bias, PD or DOD after 2 cycles and discontinuation of study arm therapy were valued also as PD or DOD after 4 cycles, respectively.

**Table 5:** Survival in molecular subgroups.

Molecular Group	Number of Patients (%)	Metastases at 1st Recurrence (%)	Median PFS_1stRD_ in Months (95%-CI)	Median OS_1stRD_ in Months (95%-CI)
WNT	2 (2.2)	2 (100)	4.1 (NA–NA)	15.7 (NA–NA)
SHH	3 (3.2)	3 (100)	2.4 (0–5.4)	2.4 (0.4–4.4)
Group 3 ^1^	16 (17.2)	15 (93.8)	4.9 (3.2–6.0)	9.8 (8.2–11.3)
Group 4 ^1^	21 (22.6)	19 (90.5)	7.2 (4.9–9.5)	20.0 (15.1–24.9)

^1^ *p*-values for the comparison of groups 3 and 4 for PFS_1stRD_ and OS_1stRD_: p_PFS_ = 0.245, p_OS_ = 0.014. NA: not applicable.

**Table 6:** PFS and OS depending on patient, disease, and treatment characteristics at first recurrence/progression. (All data are given in percentages with 95%-CI in parentheses).

Characteristics	Subgroups	2-Years PFS_1stRD_	5-Year PFS_1stRD_	10-Year PFS_1stRD_	2-Year OS_1stRD_	5-Year OS_1stRD_	10-Year OS_1stRD_
Clinical Characteristics							
Overall Cohort		19.4	9.7	7.7	38.7	15.5	8.4
		(12.8–29.3)	(5.2–18)	(3.6–16.6)	(30.0–50.0)	(9.5–25.2)	(4.1–17.4)
Sex	Male	14.9	4.5	3.0	37.3	11.9	6.0
		(8.4–26.4)	(1.5–13.5)	(0.8–11.7)	(27.4–50.9)	(6.2–22.9)	(2.3–15.4)
	Female	30.8	23.1	23.1	42.3	24.6	16.4
		(17.3–54.8)	(11.4–46.6)	(11.4–46.6)	(27.0–66.3)	(11.9–50.8)	(5.6–48.3)
Disease stage	Local	40.0	20.0	20.0	80.0	40.0	20.0
		(18.7–85.5)	(5.8–69.1)	(5.8–69.1)	(58.7–100)	(18.7–85.5)	(5.8–69.1)
	Metastatic	16.9	8.4	5.6	33.7	12.6	7.2
		(10.5–27.2)	(4.2–17.1)	(1.9–16.4)	(25.0–45.6)	(7.0–22.6)	(3.0–17.2)
Histological entity	Classical	18.4	7.9	5.9	36.8	15.4	7.7
		(11.5–29.6)	(3.7–17.0)	(2.3–15.4)	(27.4–49.5)	(9.0–26.3)	(3.4–17.4)
	Desmoplastic/nodular	30.8	23.1	23.1	61.5	23.1	23.1
		(13.6–69.5)	(8.6–62.3)	(8.6–62.3)	(40.0–94.6)	(8.6–62.3)	(8.6–62.3)
	Large cell/anaplastic	0	0	0	0	0	0
		(NA-NA)	(NA-NA)	(NA-NA)	(NA-NA)	(NA-NA)	(NA-NA)
Time-point of 1st relapse	<18 months	6.7	3.3	3.3	13.3	6.7	0
		(1.7–25.4)	(0.5–22.9)	(0.5–22.9)	(5.4–33.2)	(1.7–25.4)	(NA-NA)
	≥18 months	25.4	12.7	10.2	50.8	19.8	11.9
		(16.6–38.8)	(6.6–24.3)	(4.6–22.2)	(39.8–64.8)	(11.9–33)	(5.8–24.4)
Treatment Characteristics							
Systemic treatment	ivCHT arm	33.3	20.8	20.8	54.2	32.4	16.2
	(carboplatin/etoposide)	(18.9–58.7)	(9.6–45.4)	(9.6–45.4)	(37.5–78.3)	(18–58.4)	(6.0–43.7)
	oCHT arm	9.4	0	0	25.0	3.1	0
	(temozolomide)	(3.2–27.5)	(NA-NA)	(NA-NA)	(13.7–45.6)	(0.5–21.5)	(NA-NA)
Response to systemic chemotherapy	Objective	50.0	31.2	31.2	68.8	32.1	16.1
		(30.6–81.6)	(15.1–64.6)	(15.1–64.6)	(49.4–95.7)	(14.3–72.1)	(3.2–79.9)
	No objective	12.3	4.1	4.1	31.5	11.0	5.5
		(6.7–22.7)	(1.3–12.3)	(1.3–12.3)	(22.5–44.2)	(5.7–21.1)	(2.1–14.2)
Local treatment	Surgery	31.8	18.2	12.1	50.0	25.6	15.3
	(GTR, NTR, STR)	(17.3–58.7)	(7.5–44.1)	(3.7–40.0)	(32.9–75.9)	(12.3–53.3)	(5.5–42.8)
	No surgery/biopsy	15.5	7.0	7.0	35.2	12.1	6.0
		(9.0–26.7)	(3.0–16.4)	(3.0–16.4)	(25.7–48.3)	(6.3–23.1)	(2.2–16.9)
	Radiotherapy	50.0	30.0	22.5	55.0	33.3	26.7
		(32.3–77.5)	(15.4–58.6)	(9.4–54.1)	(37.0–81.8)	(17.5–63.5)	(12.2–58.2)
	No radiotherapy	11.0	4.1	4.1	34.2	10.8	3.6
		(5.7–21.1)	(1.4–12.4)	(1.4–12.4)	(24.9–47.1)	(5.5–21.0)	(1.0–13.4)
	Treated in	19.4	9.7	9.7	38.7	10.8	10.8
	phase II window trial	(9.4–39.7)	(3.3–28.4)	(3.3–28.4)	(24.9–60.3)	(3.5–33.4)	(3.5–33.4)

**Table 7:** Univariate and multivariate Cox regression for PFS and OS at 1^st^ recurrence.

Variable	Survival Type	Univariate Cox-Regression			Multivariate Cox-Regression		
Statistical Value		HR	95%-CI	*p*-Value	HR	95%-CI	*p*-Value
Female sex	PFS	0.56	0.34–0.94	0.03	0.83	0.46–1.49	0.52
	OS	0.70	0.42–1.15	0.16	1.01	0.46–1.59	0.62
Metastases at 1st recurrence	PFS	1.62	0.78–3.38	0.20	0.73	0.30–1.82	0.50
	OS	2.33	1.11–4.88	0.03	1.42	0.61–3.34	0.42
Desmoplastic histology	PFS	0.69	0.36–1.34	0.27	0.51	0.22–1.17	0.11
	OS	0.75	0.39–1.45	0.39	0.62	0.29–1.34	0.23
Objective response to chemotherapy at 1st recurrence	PFS	0.32	0.17–0.61	0.0005	0.23	0.10–0.55	0.0009
	OS	0.42	0.22–0.81	0.009	0.41	0.18–0.94	0.036
GTR/NTR/STR at 1st recurrence	PFS	0.56	0.33–0.97	0.04	0.72	0.37–1.39	0.32
	OS	0.60	0.35–1.04	0.07	0.91	0.48–1.73	0.78
First radiotherapy at 1st recurrence	PFS	0.31	0.13–0.73	0.002	0.12	0.04–0.38	0.0003
	OS	0.29	0.11–0.73	0.002	0.15	0.05–0.48	0.001
Time to 1st recurrence <18 months after initial diagnosis	PFS	2.34	1.47–3.73	0.0003	2.90	1.57–5.33	0.0006
	OS	3.26	2.04–5.20	<0.0001	5.97	3.02–11.79	<0.0001
Age < 6 years at 1st recurrence	PFS	0.89	0.51–1.53	0.67	0.70	0.34–1.42	0.32
	OS	1.10	0.64–1.91	0.72	0.46	0.22–0.99	0.047
Age > 16 years at 1st recurrence	PFS	1.02	0.63–1.64	0.95	0.88	0.49–1.58	0.67
	OS	0.70	0.42–1.15	0.16	0.67	0.36–1.22	0.19

**Table 8:** Acute toxicity of CTCAE grade 3 and 4 in the ivCHT arm and oCHT arm.

Study Arm	ivCHT Arm				oCHT Arm			
Cycle	1st	2nd	3rd	4th	1st	2nd	3rd	4th
	Toxicity rate (%)							
All Toxicities CTC° 3 and 4	86.4	76.1	80.9	78.9	14.3	22.6	23.1	20.8
Hematological toxicity								
Anemia	36.4	61.9	61.9	57.9	5.7	3.2	15.4	12.5
Leukopenia	68.2	61.9	80.9	68.4	2.9	9.7	15.4	16.7
Granulocytopenia	63.6	61.9	61.9	68.4	2.9	6.4	15.4	16.7
Thrombocytopenia	81.8	76.2	71.4	78.4	2.9	12.9	15.4	16.7
Non-hematological toxicity								
Infection/febrile neutropenia	50.0	33.3	38.1	26.3	2.9	6.4	11.5	0
Oral mucositis	4.5	0	0	0	0	0	0	0
Intestinal mucositis	4.5	14.2	4.8	0	0	0	0	0
Constipation	0	0	0	0	2.9	0	0	0
Skin toxicity	0	0	0	0	2.9	3.2	3.8	0
CNS neurotoxicity	4.5	4.8	4.8	5.3	2.9	6.4	0	0
Peripheral neurotoxicity	0	0	0	0	0	3.2	0	0
Nausea/Vomiting	0	0	0	0	2.9	3.2	0	0
Ototoxicity/hearing loss	4.5	4.8	9.5	10.5	5.7	3.2	3.8	4.2
Renal toxicity	0	0	0	0	0	0	0	0
Hepatotoxicity	0	0	0	0	0	0	0	0
Pulmonal toxicity	0	0	0	0	0	3.2	0	0
Cardiotoxicity	0	0	0	0	0	0	0	0

**Table 9:** Toxicity of high-dose chemotherapy.

CTC Grade	1	2	3	4
	Toxicity rate (%)			
Hematological toxicity				
Anemia	0	0	64.3	35.7
Leukopenia	0	0	0	100
Granulocytopenia	0	0	0	100
Thrombocytopenia	0	0	0	100
Non-hematological toxicity				
Infection	6.7	13.3	66.7	6.7
Febrile neutropenia	0	13.3	40.0	46.7
Oral mucositis	0	35.7	50.0	7.1
Intestinal mucositis	20.0	20.0	46.7	13.3
Skin toxicity	30.7	23.1	7.7	0
CNS neurotoxicity	11.1	11.1	0	22.2
Peripheral neurotoxicity	0	0	0	0
Nausea/Vomiting	6.7	40.0	53.3	0
Ototoxicity/hearing loss	0	20.0	80.0	0
Renal toxicity	20.0	0	0	10.0
Hepatotoxicity	41.7	25.0	33.3	0
Pulmonary toxicity	0	20.0	30.0	0
Cardiotoxicity	0	30.0	0	0

## Data Availability

The data presented in this study are available on request from the corresponding author.
